# Betulinic acid induces apoptosis and inhibits hedgehog signalling in rhabdomyosarcoma

**DOI:** 10.1038/sj.bjc.6605715

**Published:** 2010-06-01

**Authors:** M Eichenmüller, B Hemmerlein, D von Schweinitz, R Kappler

**Affiliations:** 1 Department of Paediatric Surgery, Dr. von Hauner Children's Hospital, Ludwig-Maximilians-University Munich, Lindwurmstrasse 4, Munich 80337, Federal Republic of Germany; 2 Department of Pathology, Georg-August-University Goettingen, Robert-Koch-Strasse 40, Goettingen 37075, Federal Republic of Germany

**Keywords:** betulinic acid, rhabdomyosarcoma, apoptosis, hedgehog, xenograft

## Abstract

**Background:**

Rhabdomyosarcoma (RMS) is the most common soft-tissue sarcoma in childhood with the ability to resist apoptosis by the activation of survival promoting and anti-apoptotic proteins.

**Methods:**

Efficacy of the apoptosis-inducing agent betulinic acid (BA) was determined in RMS cell cultures and *in vivo* by measuring cell viability, survival, apoptosis, hedgehog signalling activity, and neovascularisation.

**Results:**

Betulinic acid had a strong cytotoxic effect on RMS cells in a dose-dependent manner. The BA treatment caused a massive induction of apoptosis mediated by the intrinsic mitochondrial pathway, which could be inhibited by the broad-range caspase inhibitor zVAD.fmk. Exposure of hedgehog-activated RMS-13 cells to BA resulted in a strong decrease in *GLI1*, *GLI2*, *PTCH1*, and *IGF2* expression as well as hedgehog-responsive luciferase activity. Intraperitoneal injection of 20 mg BA per kg per day significantly retarded growth of RMS-13 xenografts in association with markedly higher counts of apoptotic cells and down-regulation of GLI1 expression compared with control tumours, while leaving microvascular density, cell proliferation, and myogenic differentiation unaffected.

**Conclusion:**

Our data show that induction of apoptosis and inhibition of hedgehog signalling are important features of the anti-tumourigenic effect of BA in RMS and advices this compound for the use in a multimodal therapy of this highly aggressive paediatric tumour.

Rhabdomyosarcoma (RMS) is the most common soft-tissue sarcoma in children, representing ∼9% of paediatric solid cancers (Crist and Kun, 1991). This tumour is thought to arise from skeletal muscle precursor cells by interruption of normal myogenic proliferation and differentiation processes, which are tightly regulated through a variety of developmental genes, external growth factors, and cell cycle-associated genes (Merlino and Helman, 1999). Rhabdomyosarcoma is characterised by having two major subtypes that differ in histology, degree of differentiation, and prognosis: the embryonal form (ERMS) constituting approximately two-thirds of all RMS and the rarer alveolar form (ARMS) (Newton *et al*, 1995). Both subtypes are also distinguishable based on the detection of distinct molecular/genetic markers. The ERMS is generally characterised by loss of heterozygosity at the chromosomal band 11p15 (Visser *et al*, 1997), a region harbouring the imprinted genes *IGF2* and *H19*. In contrast, the majority of ARMS are reported to have the characteristic reciprocal translocations t(2;13)(p35;q14) and t(1;13)(p36;q14), giving rise to PAX3– and PAX7–FKHR fusion proteins, respectively (Shapiro *et al*, 1993; Davis *et al*, 1994). Currently, there is data to suggest that ARMS are less sensitive to therapeutic modalities than ERMS, with estimated 3-year event-free survival rates of 66% and 83%, respectively (Crist *et al*, 2001). Furthermore, high mortality rates remain in patients with metastatic disease (Breneman *et al*, 2003). Failure of RMS treatment is commonly considered on the basis of resistance of cancer cells to chemotherapeutic drugs. Recent studies, including those from our laboratory, have shown that RMS characteristically display activation of cell survival promoting and anti-apoptotic proteins such as Akt and Bcl-2 (Kappler *et al*, 2003; Armistead *et al*, 2007). This suggests that the resistance of RMS to chemotherapeutic drugs might be associated with the inability of tumour cells to undergo apoptosis. Therefore, an innovative therapeutic approach using restoration of apoptotic defence might be effective for the treatment of patients with this highly malignant tumour.

The naturally occurring pentacyclic triterpenoid betulinic acid (BA) has been shown to possess anti-tumoural activity and overcome resistance by inducing apoptosis in a variety of human cancers (for review, see Alakurtti *et al*, 2006). Its selective cytotoxicity against cancer was first described on human melanoma both *in vitro* and *in vivo* (Pisha *et al*, 1995). Since that initial study, BA has been reported to be effective on a growing number of human cancers, including those of the lung, colon, prostate, and ovary (Alakurtti *et al*, 2006), whereas normal cells were unaffected by BA treatment (Zuco *et al*, 2002). Interestingly, BA has also been successfully applied *in vitro* in childhood cancers, namely medulloblastoma, glioblastoma, Ewing sarcoma (Fulda *et al*, 1999), neuroblastoma (Schmidt *et al*, 1997), and leukaemia (Ehrhardt *et al*, 2004). Accumulated experimental evidence indicates that BA causes distinct morphological changes in sensitive cells, such as cell shrinkage, DNA fragmentation, nuclear condensation, and membrane blebbing (Alakurtti *et al*, 2006). The exact molecular mechanisms underlying BA-induced apoptosis are still not clear, but several studies suggest that the proteolytic cleavage of caspases, the activation of the MAP kinase cascade, the modulation of NF-κB signalling, the generation of reactive oxygen species, and the inhibition of topoisomerase I may have a function therein (for review, see Alakurtti *et al*, 2006). In addition, BA has been shown to be effective on both drug-resistant and drug-sensitive tumour cell lines, showing its significant therapeutic benefit to chemotherapy-resistant cancer patients (Sarek *et al*, 2003).

As BA suppresses tumour growth by inducing apoptosis, we hypothesised that this phytochemical might promote cell death in human RMS cells. In this study, we have investigated the time- and dose-dependent growth inhibitory effects of BA in three RMS cell lines and scrutinised whether BA treatment could lead to the induction of apoptosis by the mitochondrial pathway. In addition, we determined the impact of BA on hedgehog signalling, which has been implicated in RMS development (Kappler *et al*, 2003; Tostar *et al*, 2006). As *in vivo* data on BA treatment are scarce, we furthermore analysed the *in vivo* effects triggered by BA in RMS-13 xenografts.

## Materials and methods

### Cell lines

The human RMS cell lines RH-30, RMS-13, and RD, as well as mouse NIH-3T3 fibroblasts, were purchased from the German Collection of Microorganisms and Cell Cultures (DSMZ, Braunschweig, Germany) and the American Type Culture Collection (Manassas, VA, USA). All cell lines were maintained as the suppliers recommended and tested for authentication purposes for the expression of the myogenic markers MyoD1, Myogenin, and Desmin (data not shown).

### Cell viability and morphology

Cell growth was assessed using the Cell Proliferation Kit I (Roche Diagnostics, Penzberg, Germany) according to the manufacturer's protocol. Cells were seeded at a density of 5 × 10^3^ cells per 96-well plate (Nunc, Wiesbaden, Germany), and after overnight attachment, cells were treated for 0–72 h with 0–50 *μ*g ml^–1^ BA (BioSolutions, Halle, Germany) dissolved in DMSO. Morphological changes of incubated cells were documented with a Zeiss-inverted phase-contrast microscope equipped with a Canon PowerShot G6 digital device. Cell viability was measured after addition of 3-(4,5-dimethylthiazol-2-yl)-2,5-diphenyltetrazolium bromide (MTT) labelling reagent on the GENios reader (Tecan, Männedorf, Switzerland) at a wavelength of 595 nm.

### Clonogenic assay

Cells were seeded at a density of 2 × 10^3^ cells per six-well plate. After overnight attachment, cells were treated with 0–10 *μ*g ml^–1^ BA for 48 h. After further 8 days growth without any treatment, cells were fixed with methanol for 5 min and stained with 0.5% crystal violet. Clonogenic survival was determined by counting stained colonies under a Zeiss-inverted phase-contrast microscope and equipped with a Canon PowerShot G6 digital device.

### Flow cytometric analysis

Cells were seeded at a density of 5 × 10^4^ cells per 24-well plate. After overnight attachment, cells were treated for 48 h with 0–10 *μ*g ml^–1^ BA in the presence or absence of 50 *μ*M of the broad-range caspase inhibitor zVAD.fmk (Bachem AG, Bubendorf, Schweiz) dissolved in DMSO. Cells were trypsinised, washed in PBS, and resuspended in 3.4 mM sodium citrate per 0.1% Triton-X 100. Apoptotic cells were detected as a reduced sub-G_1_ peak (fragmented DNA) after propidium iodide staining (1 mg ml^–1^) using an FACscan (Becton Dickinson, Heidelberg, Germany).

### Western blot analysis

Cells were seeded at a density of 2 × 10^5^ cells per six-well plate, and after overnight attachment, cells were treated for 48 h with 0–10 *μ*g ml^–1^ BA in the presence or absence of 50 *μ*M zVAD.fmk. Cells treated with vehicle alone (DMSO) or 100 *μ*g ml^–1^ cycloheximide (Sigma-Aldrich, Taufkirchen, Germany) were used as negative and positive control, respectively. After treatment, cells were scraped and lysed in PBS per 0.5% Triton-X 100 per 1 mM sodium-orthovanadate. Protein lysates were cleared of cellular debris by centrifugation for 5 min at 13 000 r.p.m. at 4°C. The protein concentration was determined by the Protein Assay System (Bio-Rad, Hercules, CA, USA). Equivalent amounts of proteins were separated on 12% SDS–PAGE under reducing conditions and then transferred to nitrocellulose membranes (GE Healthcare, Piscataway, NJ, USA). The membranes were incubated with PBS per 0.1% Tween 20 and 5% non-fat dry milk to block non-specific binding. Membranes were incubated for 1.5 h with mouse anti-human caspase 3, rabbit anti-human cleaved caspase 3, rabbit anti-human poly(ADP-ribose) polymerase (PARP), rabbit anti-human *β*-actin (all from Cell Signalling Technology, Danvers, MA, USA), mouse anti-human COX4, rabbit anti-human cytochrome c (both from Clontech, Mountain View, CA, USA) or goat anti-human GLI1 (C18; Santa Cruz Biotechnology, Santa Cruz, CA, USA) antibodies, and thereafter for 1 h with horseradish peroxidase-conjugated goat anti-rabbit, goat anti-mouse, or rabbit anti-goat IgG secondary antibodies (all from DakoCytomation, Hamburg, Germany). Signals were visualised using the ECL chemiluminescence detection system (GE Healthcare).

### Measurement of mitochondrial transmembrane potential

Cells were seeded at a density of 5 × 10^4^ cells per 24-well plate. After overnight attachment, cells were treated for 2–18 h with 10 *μ*g ml^–1^ BA. Cells were then trypsinised, washed in PBS, incubated for 15 min with 40 nM of the cationic lipophilic fluorochrome 3,3′-dihexyloxacarbocyanide iodide (DiOC_6_(3); Molecular Probes, Eugene, OR, USA) at 37°C and subsequently analysed by flow cytometry using an FACscan (Becton Dickinson).

### Real-time RT–PCR

Total RNA was extracted from tumour cell lines in Trizol (Invitrogen, Carlsbad, CA, USA) according to the manufacturer's instructions. Reverse transcription of total RNA was performed using random hexamers (Roche Diagnostics) and SuperScriptII reverse transcriptase (Invitrogen). Real-time PCR and quantisation of gene expression was performed as described earlier (Eichenmuller *et al*, 2009a). For the human genes, we used the following primer pairs (5′ → 3′ orientation): *GLI1*, AGCTACATCAACTCCGGCCA, GCTGCGGCGTTCAAGAGA; *GLI2*, TTCTCCAACGCCTCGGAC, GCCTGGGATCTTGCAGATGT; *PTCH1*, TTGATTGTGGGTGGCACAGT, GCTTGGGAGTCATTAACTGGAAC; *IGF2*, CCTCCGACCGTGCTTCC, GGTGGACTGCTTCCAGGTGT; *TBP*, GCCCGAAACGCCGAATAT, CCGTGGTTCGTGGCTCTCT. For the murine genes, we used the following primer pairs: *Shh*, GGGAGCAGACCGGCTGAT, CAGCTTCACTCCAGGCCACT; *Gli1*, GAATCGGACCCACTCCAATG, GTGTTTGCGGAGCGAGCT; *Gli2*, GTCACTGAAGGATTCCTGCTCG, CAGGACAGAACCATTGACTGGA; *Ptch1*, CTCCTGAAACCCAAAGCCAA, CGTCTCTCACTCGGGTGGTC; *Igf2*, CGGAGAGACTCTGTGCGGA, ACGGCTTGAAGGCCTGC; *Tbp*, TCACTCCTGCCACACCAGC, TCAAGTTTACAGCCAAGATTCACG. Amplification of the house-keeping gene *TATA-Box-binding-Protein* (*TBP*, *Tbp*) was performed to standardise the amount of sample RNA.

### Hedgehog reporter and activation assays

A total of 5 × 10^4^ RMS-13 cells or NIH-3T3 fibroblasts were seeded in 12-well plates the day before transfection. Tumour cells were then transfected with 900 ng of the reporter plasmid p11xGli or the empty control pGL3-TK (Beer *et al*, 2003) and 100 ng of the reference plasmid pRL-TK using FuGene 6 transfection reagent (Roche Diagnostics). Fibroblasts were accordingly transfected with 1 *μ*g of an expression plasmid containing the entire open reading frame of the murine sonic hedgehog gene, which was kindly supplied by Dr R Toftgard (Karolinska Institute, Stockholm, Sweden). Twenty-four hours after transfection, cells were supplemented with 10 *μ*g ml^–1^ BA, 7.5 *μ*M cyclopamine (Toronto Research Chemicals, Toronto, Canada), or vehicle as indicated and cultured for 24 h. For reporter assay, cells were lysed and reporter gene activity was determined using the Dual-Glo Luciferase Reporter Assay System (Promega, Madison, Wisconsin, USA). Firefly luciferase activity was normalised to Renilla luciferase activity. All reporter assay experiments were repeated at least three times and transfections performed in duplicate. For gene expression analysis, total RNA was isolated from fibroblasts 24 h after transfection using Trizol (Invitrogen).

### Xenograft assay

Female NMRI nude mice (6–8 weeks old) were purchased from Charles River (Sulzfeld, Germany) and housed in accordance with the Institutional Animal Care guidelines. This study was approved by the District Government of Upper Bavaria. Mice were divided into two groups of five mice. Exponentially growing RMS-13 cells were detached from culturing dishes by scraping and a 0.1-ml suspension containing 2 × 10^7^ cells was injected s.c. on the right flanks above the hindlimb of each mouse. After 1 week, first tumours were visible and palpable. After 10 days of tumour cell injection, mice were treated intraperitoneally with a dose of 40 mg BA per kg body weight or vehicle every second day for a total period of 16 days. The dose of BA was decided by referring to an earlier report, which described a significant anti-tumour activity at doses of 20 mg kg^–1^ per day and lack of toxicity at repeated doses up to 500 mg kg^–1^ (Pisha *et al*, 1995). Tumour size was measured every other day using calliper, and tumour volume was calculated using the formula (*X*/2) × (*Y*/2) × (*Z*/2) × (4/3 × 3.1415). Control mice were injected with 0.1 ml vehicle with a similar dosing schedule. Body weights of the control and BA-treated mice were recorded throughout the experiment. Mice were also monitored for other symptoms of side effects including food and water withdrawal and impaired posture or movement.

### Immunohistochemistry

Tumour tissues from control and BA-treated mice were fixed in 4% neutral-buffered formalin, dehydrated, embedded in paraffin, and sectioned at 4 *μ*m thickness. Cross-sections were deparaffinised and rehydrated, and haematoxylin and eosin or Gomori's trichrome stained for histopathologic evaluation. For immunohistochemistry, endogenous peroxidase activity was quenched with 3% hydrogen peroxide in bi-distilled water. For antigen retrieval, sections were either heated in a steamer in citrate buffer (pH6) for 40 min or digested with protease XXIV (Sigma, Munich, Germany) at 37°C for 15 min. Sections were then incubated overnight with a polyclonal rabbit anti-cleaved caspase 3 (Cell Signalling Technology), monoclonal mouse anti-Ki-67 (BD PharMingen, San Diego, CA, USA), rat anti-mouse CD31 (Acris, Herford, Germany), mouse anti-rat myogenin (DakoCytomation), or polyclonal rabbit anti-GLI1 (H-300; Santa Cruz Biotechnology) antibody all diluted 1 : 50 in PBS to assess apoptosis, cell proliferation, microvessel density, myogenic differentiation, and hedgehog signalling, respectively. Signal detection was achieved using the Envision-Peroxidase system (DakoCytomation) with diaminobenzidine as chromogen. Tumour sections were counterstained with haematoxylin. For the quantification of mean microvessel density, total transverse sections were stained for CD31 and evaluated in 10 randomly selected fields at × 400 magnification.

### Statistical analysis

Data were expressed as means±s.d. and statistically subjected to Student's unpaired *t*-test. A level of *P*<0.05 was considered to be significant.

## Results

### BA treatment causes reduced cell viability and survival of RMS cells

**Figure 1:**
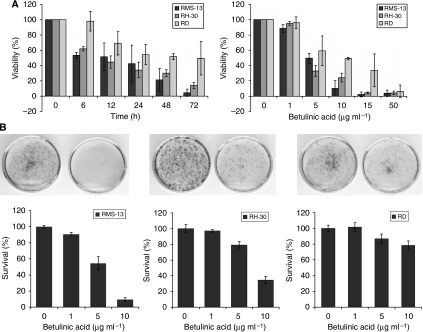
(**A**) Decreased viability of RMS cells on BA treatment. The RMS cell lines were treated for 0–72 h with 10 *μ*g ml^–1^ (left panel) and for 48 h with 0–50 *μ*g ml^–1^ (right panel) BA. Cell viability was analysed by MTT assay in three independent experiments and is depicted as the mean percentage±s.d. of viable cells. (**B**) BA inhibits clonogenic survival. The RMS cell lines were treated for 48 h with 0–10 *μ*g ml^–1^ BA. Representative experiments of untreated (left) and 10 *μ*g ml^–1^ BA-treated (right) cells are shown. Clonogenic survival was measured by counting crystal violet-stained colonies after 8 days. Mean and s.d. of triplicates are shown; similar results were obtained in two independent experiments.

Owing to the remarkable success that BA displayed against many forms of human cancer *in vitro*, we decided to investigate its effects on RMS over time (0–72 h) and selected a BA concentration comparable with the one used in earlier reported studies (Pisha *et al*, 1995; Fulda *et al*, 1997). As depicted in Figure 1A (left panel), 10 *μ*g ml^–1^ BA resulted in a 40% loss of viable cells in RH-30 and RMS-13 cells already after 6 h, as measured by MTT assay. At the end of the time course (72 h), viability of both cell lines was <20% by this BA concentration. However, RD cells display a certain adaptation to BA treatment over time. In a next step, we determined the effect of different BA concentrations (0–50 *μ*g ml^–1^) on RMS cells for a treatment period of 48 h. We observed that BA influences RMS-13 and RH-30 cells in a dose-dependent manner, whereas RD cells were less responsive against BA treatment (Figure 1A, right panel). However, high micromolar concentrations (50 *μ*g ml^–1^) resulted in complete killing of all cell lines. The half-maximal cytotoxic concentration (IC_50_) of BA was 5.0, 3.9, and 9.5 *μ*g ml^–1^ for RMS-13, RH-30, and RD, respectively. To scrutinise long-term effects of BA on RMS cells, clonogenic assays were performed after adding varied concentrations of BA (0–10 *μ*g ml^–1^). Loss of clonogenic survival of 90% and 65% was shown at 10 *μ*g ml^–1^ BA in RMS-13 and RH-30 (Figure 1B), respectively. In contrast, clonogenic survival of RD cells was hardly reduced after BA treatment, indicating that these cells are less responsive to BA.

### BA induces apoptosis in RMS cells by affecting mitochondria

**Figure 2:**
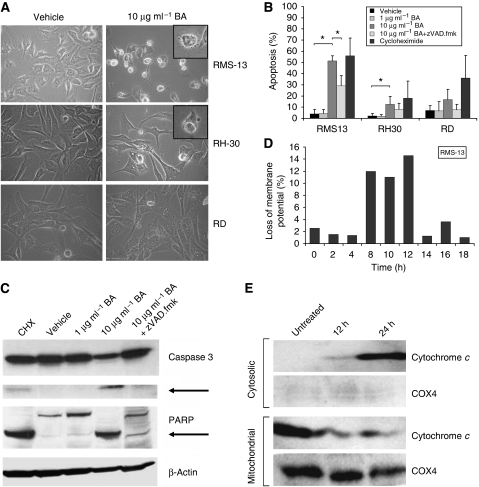
BA-induced apoptosis in RMS cell lines. (**A**) Morphology. The RMS cells were treated for 48 h with or without 10 *μ*g ml^–1^ BA. Typical morphological changes after apoptosis induction were observed in RMS-13 and RH-30 cells, but not in RD cells. Insets show apoptotic cells at high magnification. (**B**) DNA fragmentation. The RMS cells were treated for 48 h with 0–10 *μ*g ml^–1^ BA in the presence or absence of 50 *μ*M of the broad-range caspase inhibitor zVAD.fmk. Apoptosis-specific DNA fragmentation was determined by FACS analysis of propidium iodide-stained nuclei; 100 *μ*g ml^–1^ cycloheximide (CHX) was used as a positive control for apoptosis induction. Mean and s.d. from three independent experiments are shown; *P<0.05 (unpaired Student's *t*-test). (**C**) Activation of apoptosis-specific proteins. The RMS-13 cells were treated for 48 h with 0–10 *μ*g ml^–1^ BA in the absence or presence of the broad-range caspase inhibitor zVAD.fmk. Expression of caspase 3, PARP, and *β*-actin was detected by western blot analysis. Cleaved caspase 3 and PARP products are indicated by arrows; 100 *μ*g ml^–1^ cycloheximide (CHX) was used as a positive control for apoptosis induction. (**D**) Loss of mitochondrial membrane potential. The RMS-13 cells were treated for 0–18 h with 10 *μ*g ml^–1^ BA. Mitochondrial membrane potential was measured by flow cytometry using the potential-sensitive fluorochrome DiOC_6_(3). (**E**) Cytochrome c release. The RMS-13 cells were treated for 12 and 24 h with 10 *μ*g ml^–1^ BA. Mitochondria were isolated, lysed, and the expression of cytochrome c and COX4 in the mitochondrial and cytosolic fraction was detected by western blot analysis.

As BA exerts its anti-tumourigenic effect by inducing apoptosis, we next examined whether BA can induce morphological changes in RMS. We found that BA treatment resulted in cell shrinkage, membrane blebbing, and nuclear fragmentation of RMS-13 and RH-30 cells (Figure 2A). In contrast, these apoptotic features were not evident in RD cells. We next assessed BA-induced apoptosis by flow cytometry of propidium iodide-stained nuclei (Figure 2B). The DNA fragmentation was mainly detected in RMS-13 and to a lower percentage in RH-30 and RD cells 48 h after BA treatment. However, in the presence of the broad-range caspase inhibitor zVAD.fmk BA-induced apoptosis was significantly reduced in RMS-13 cells, and to a lesser extent in RH-30 and RD cells (Figure 2B), indicating that caspases were involved in BA-induced apoptosis. We then selected the most responsive RMS cell line, RMS-13, to see whether BA-induced apoptosis indeed requires caspase activation. Therefore, we monitored the cleavage of caspase 3 by western blot analysis 48 h after BA treatment (Figure 2C). Incubation of RMS-13 cells with BA led to cleavage of caspase 3 and its known downstream target PARP. This effect was partially blocked in the presence of zVAD.fmk, thus providing evidence that the caspase-dependent pathway is involved in BA-induced apoptosis.

As caspases act both in the extrinsic pathway (mediated by death receptors) and the intrinsic pathway (mediated by death signals inducing mitochondrial proteins), we wanted to know which pathway is involved in BA-induced apoptosis of RMS. Analysis of active caspase 8, which is a marker for the extrinsic pathway, showed no changes after BA treatment (data not shown). Conversely, by studying the effect of BA on mitochondrial function, we found that incubation with BA results in an enhanced breakdown of mitochondrial membrane potential in RMS-13 cells (Figure 2D), reaching a maximum between 8 and 12 h after BA treatment. Next, we analysed whether this effect on mitochondria could lead to the release of cytochrome c protein into the cytosol. Our western blot analysis revealed increased cytochrome c levels in the cytosol already 12 h and more pronounced 24 h after BA treatment, whereas the cytochrome c concentration in the mitochondrial fraction inversely decreased (Figure 2E). Together, these results clearly indicate that BA-induced apoptosis in RMS-13 cells is mediated by the intrinsic apoptotic pathway.

### BA specifically targets hedgehog signalling in RMS cells

**Figure 3:**
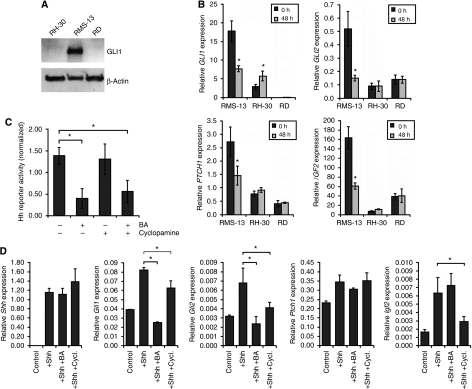
BA blocks hedgehog signalling (**A**) GLI1 expression of rhabdomyosarcoma cell lines. The RMS cell lines were lysed and the expression of GLI1 and *β*-actin was detected by western blot analysis. (**B**) Hedgehog signalling dependency. The RMS cells were treated for 48 h with 10 *μ*g ml^–1^ BA. *GLI1*, *GLI2*, *PTCH1*, and *IGF2* mRNA expression from untreated (black bars) and treated (grey bars) RMS cells was measured by quantitative real-time PCR in relation to the house-keeping gene *TBP* as a calibrator; *P<0.05 (unpaired Student's *t*-test). (**C**) Hedgehog pathway activity. The RMS-13 cells were transiently transfected with 900 ng hedgehog-responsive reporter plasmid (p11xGli) or control plasmid (pGL3-TK) and treated for 24 h with 10 *μ*g ml^–1^ BA, 7.5 *μ*M cyclopamine, 10 *μ*g ml^–1^ BA, and 7.5 *μ*M cyclopamine, or vehicle. Firefly luciferase activity was measured and normalised to the subsequently measured Renilla luciferase activity. Reporter assay experiments were repeated three times and transfections performed in duplicate; *P<0.05 (unpaired Student's *t*-test). (**D**) Hedgehog activation assay. The NIH-3T3 cells were transiently transfected with 1 *μ*g sonic hedgehog (Shh) expression plasmid. For the inhibition of hedgehog signalling, cells were additionally exposed to 10 *μ*g ml^–1^ BA and 7.5 *μ*M cyclopamine (Cycl.) 24 h after transfection. Expression of the murine genes *Shh*, *Gli1*, *Gli2*, *Ptch1*, and *Igf2* was determined after an incubation period of 48 h using quantitative real-time PCR in relation to the house-keeping gene *Tbp* as a calibrator; *P<0.05 (unpaired Student's *t*-test).

Inappropriate hedgehog signalling has a fundamental function in an increasing number of malignancies, including childhood solid tumours such as medulloblastoma and RMS (Evangelista *et al*, 2006). Interestingly, the most BA-responsive RMS cell line, RMS-13, is known to display an activated hedgehog pathway caused by a massive DNA amplification of the *GLI1* locus (Roberts *et al*, 1989). Thus, we wondered whether BA has any impact on this signalling cascade. First, we verified that this genomic alteration results in a strong expression of the GLI1 protein in RMS-13 cells by western blot analysis (Figure 3A). Next, we analysed the expression of the known hedgehog target genes *GLI1*, *GLI2*, *PTCH1*, and *IGF2* in RMS-13 cells after BA treatment. Strikingly, we found a significant reduction of mRNA level for all four genes in RMS-13 cells treated with BA (Figure 3B). This contrasts the situation found in the two GLI1-negative RMS cell lines RH-30 and RD (Figure 3A), as transcription of these genes stayed grossly unchanged (Figure 3B). Moreover, we found a significant reduction in hedgehog activity of RMS-13 cells after BA treatment by means of a hedgehog-responsive reporter assay (Figure 3C). To examine whether this inhibition is dependent on hedgehog signalling components upstream of GLI1, RMS-13 cells were treated with BA in the presence or absence of 7.5 *μ*M cyclopamine, a specific hedgehog signalling inhibitor (Taipale *et al*, 2000). However, the inhibitory effect of BA on GLI1 activity was found to be independent of cyclopamine treatment (Figure 3C), indicating that BA is able to selectively target hedgehog signalling on the level of GLI1.

To further elucidate the concept of downstream hedgehog pathway inhibition by BA under more physiological conditions, we made use of untransformed NIH-3T3 fibroblasts, which have been described to be responsible to sonic hedgehog (Taipale *et al*, 2000). Activation of the pathway in NIH-3T3 cells by expressing the ligand sonic hedgehog on transient transfection resulted in a strong increase in expression of the known hedgehog target genes *Gli1*, *Gli2*, *Ptch1*, and *Igf2* (Figure 3D). As expected, the known hedgehog inhibitor cyclopamine led to a significant reduction of target gene expression in this activation assay. Strikingly, BA treatment had an even stronger inhibitory effect on *Gli1* and *Gli2* expression, whereas *Ptch1* and *Igf2* stayed grossly unchanged. Altogether, these findings indicate that BA is able to selectively target hedgehog signalling directly on the level of GLI family transcription factors.

### Intraperitoneally administration of BA inhibits RMS-13 xenograft growth in athymic nude mice by selectively inducing apoptosis

**Figure 4:**
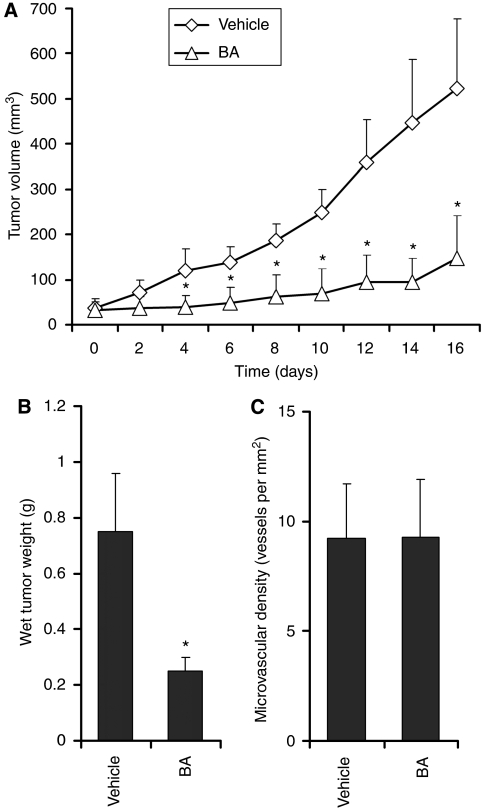
BA treatment suppressed RMS-13 xenograft growth. Female NMRI nude mice carrying RMS-13 xenograft tumours were treated intraperitoneally for 16 days with 20 mg BA per kg body weight per day or vehicle. (**A**) BA treatment induced a statistically significant decrease in tumour growth as determined by volumetric measurement of BA-treated (*n*=5) and vehicle-treated (*n*=5) mice. Error bars represent standard error of the mean; *P<0.05 (unpaired Student's *t*-test). (**B**) Mean tumour weight and standard error of freshly dissected tumours of BA-treated and vehicle-treated mice are shown; *P<0.05 (unpaired Student's *t*-test). (**C**) Mean microvascular density was determined by counting CD31-immunostained vessels per mm^2^ in RMS-13 xenografts of vehicle or BA-treated mice. Error bars represent standard error of the mean.

Next, we designed animal experiments to test whether BA administration effectively inhibits growth of RMS-13 xenografts and if BA causes programmed cell death in tumours *in vivo*. To reach this, we treated a group of mice (*n*=5) with a palpable tumour mass of ∼50 mm^3^ with 40 mg BA per kg every second day for a total of eight doses and compared their tumour growth with a vehicle-treated control group (*n*=5). As shown in Figure 4A, BA treatment resulted in a dramatic reduction in tumour growth compared with controls, as determined by measuring tumour volume over time. However, tumour regression was not observed. Furthermore, the lowered tumour growth was accompanied by significantly decreased tumour weights in the treatment group (Figure 4B). As it has been described that BA has anti-angiogenetic effects on prostatic tumours in mice (Chintharlapalli *et al*, 2007), we next evaluated blood microvessel density to see whether lowered blood supply could be the reason for the growth suppression of BA. In contrast to Chintharlapalli and co-workers, our extensive morphometric analysis revealed no effects of BA treatment on blood microvessel density (Figure 4C).

**Figure 5:**
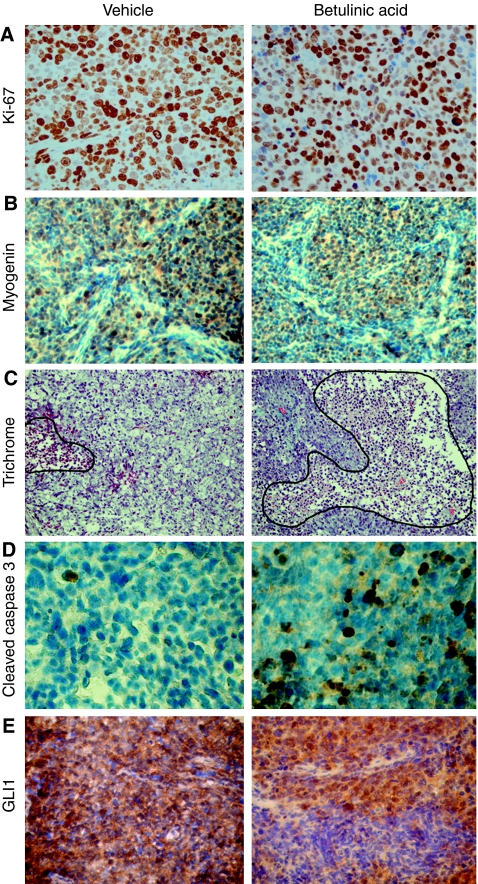
Induction of apoptosis and hedgehog inhibition in RMS *in vivo*. Representative photographs of histological and immunohistochemical stainings of untreated (left) and BA-treated (right) RMS-13 xenografts. (**A**) Comparable amounts of cell proliferation in BA-treated and untreated tumours as revealed by Ki-67 immunostaining. (**B**) Lack of induced differentiation on BA treatment was revealed by immunostaining of the late muscle marker myogenin. (**C**) Trichrome staining depicting extensive tumour destruction (hemmed by a black line) in tumours of the treatment group. (**D**) Cleaved caspase 3-immunopositive cells indicate a significant higher apoptotic rate in BA-treated tumours compared with controls. (**E**) Lack of GLI1 staining in large areas of BA-treated xenografts indicates suppression of hedgehog signalling.

To investigate whether alteration in cell proliferation and/or differentiation may have caused growth suppression of RMS-13 xenografts on BA treatment, we have stained for appropriate markers by using immunohistochemistry. Unexpectedly, we found no differences in the proportion of Ki-67 (Figure 5A) and Myogenin-positive (Figure 5B) cells between BA- and vehicle-treated tumours. However, histological examination of tumours of the BA-treatment group revealed extensive tumour destruction as evidenced by large areas of necrosis (Figure 5C). In line with this, we detected a significant increase of apoptosis in BA-treated tumours compared with the control group, as evidenced by staining for the apoptotic marker cleaved caspase 3 (Figure 5D). Strikingly, BA treatment led to a more regional expression of GLI1 leaving large tumour areas unstained, as compared with the homogeneous GLI1 staining in untreated tumours. This suggests at least a partial down-regulation of the hedgehog target on BA treatment. Altogether, these findings indicate that BA is a potent inhibitor of RMS-13 growth *in vivo* by selectively activating pro-apoptotic mechanisms and inhibiting hedgehog signalling, without affecting the proliferative, differentiating, and angiogenetic properties of this tumour.

## Discussion

Despite the development of multimodal therapies, the overall cure rate of RMS remains low (Crist *et al*, 2001; Breneman *et al*, 2003). Failure of RMS treatment is commonly considered on the basis of resistance of cancer cells to chemotherapeutic drugs. Thus, the development and validation of new therapeutic approaches is of utmost importance. This study revealed that BA administration might be a promising strategy, as it not only inhibits growth of human RMS cells by inducing apoptosis, but also blocks the hedgehog signalling pathway.

There is accumulated experimental evidence indicating that BA-induced apoptosis is not mediated by the extrinsic CD95 or other death receptor/ligand systems, but acts directly on the intrinsic mitochondrial pathway (Fulda *et al*, 1997, 1998). From our data, it is safe to predict that BA induces apoptosis in RMS cells too through the mitochondrial pathway, as evidenced by loss of mitochondrial membrane potential, cytochrome c release from mitochondria, increased levels of proteolytically cleaved caspase 3 and PARP, as well as absence of caspase 8 activation. Thus, by directly initiating mitochondria-dependent apoptosis and thereby acting on a more downstream level of apoptosis control BA administration might be advantageous for treating tumours such as RMS in which upstream components of apoptosis-inducing signalling pathways are interrupted. One of these components, the tumour suppressor protein p53, has been implicated in the cellular response to stress such as DNA damage, hypoxia, and oncogene activation and mediates either growth arrest or apoptosis depending on cell type and particular conditions (Vousden and Lu, 2002). However, as p53 wild-type (RMS-13; Muller *et al*, 2007) and p53 mutant RMS cells (RH-30 and RD; Barlow *et al*, 2005) were sensitive to BA treatment, it might be assumed that BA-induced apoptosis in RMS cells is mediated by p53-independent pathways. This is in line with an earlier study reporting lack of wild-type p53 accumulation on BA treatment as well as association between p53 mutation status and BA sensitivity in another paediatric cancer, namely neuroblastoma (Fulda *et al*, 1997). Together, these data suggest that BA induces apoptosis in RMS through the mitochondrial pathway in a p53-independent manner.

Angiogenesis is generally accepted as a prerequisite for tumour growth beyond certain size as well as for metastatic spread and, therefore, represents an attractive new target for tumour therapy (Folkman, 2002). In recent years, anti-angiogenic treatments have been shown to synergise with traditional chemotherapeutic and radiotherapeutic regimens. Of note, BA has been described to have not only a cytotoxic effect on endothelial cells, but also inhibits tube-like structure formation of aortic endothelial cells (Kwon *et al*, 2002; Mukherjee *et al*, 2004). However, using extensive morphometric analyses, our study clearly shows that the growth-inhibiting effect in RMS is solely based on the increase of apoptosis in BA-treated tumours leaving CD31-positive microvessel density, Ki-67-based proliferation index, and differentiation status unaffected. This contrasts a recent study by Chintharlapalli *et al* (2007) who reported on BA-induced anti-angiogenetic effects in prostatic cancer cells grown as xenografts in nude mice. The discrepancy in data concerning the anti-angiogenetic effect of BA may be explained by the presence of alternative angiogenic factors in RMS other than vascular endothelial growth factor (VEGF), which has been described to be the primary target during BA-induced anti-angiogenesis in LNCaP prostatic xenografts (Chintharlapalli *et al*, 2007). The RMS are known to produce basic fibroblast growth factor and interleukin-8 that stimulate their growth and neovascularisation (Schweigerer *et al*, 1987; Pavlakovic *et al*, 2001). Accordingly, Zhang *et al* (2002) showed that monoclonal anti-VEGFR2 antibody treatment of RMS xenografts led only to minor effects on tumour growth and angiogenesis, but when combined with low-dose, doxorubicin results in full responsiveness and complete remission. Contrarily, antibody-mediated inhibition of VEGF in prostate cancer led to complete suppression of angiogenesis and prevents tumour growth beyond the initial prevascular growth phase (Borgstrom *et al*, 1998). Thus, the redundancy of angiogenesis-sustaining factors in RMS could be responsible for the high degree of vascularity found in our treatment group after BA administration.

Another intriguing finding of our study is that BA impacts on the hedgehog signal transduction pathway, which is known to be aberrantly activated in a variety of human cancers, including RMS (Kappler *et al*, 2003; Tostar *et al*, 2006). Overproduction of hedgehog ligands, gain-of-function mutations in *smoothened*, or loss-of-function mutations in *patched* (*PTCH1*) and *suppressor of fused*, all leading to constitutively activation of GLI family transcription factors are the generally anticipated cause for inappropriate hedgehog activation (reviewed in Evangelista *et al*, 2006). Thus, specific down-regulation of GLI family transcription factors at the distal end of the signalling pathway might be a desirable approach for bypassing the likely myriad of upstream activating events. Our study now shows that BA is not only effective on RMS-13 cells by decreasing cell viability and inducing apoptosis, but also by effectively suppressing hedgehog target gene expression. This is in line with our earlier finding that BA suppresses hedgehog target genes in hepatoblastoma, another embryonal tumour of early childhood (Eichenmuller *et al*, 2009b) and a study by Rzeski *et al* (2006), which recently described that BA decreases expression of *BCL2*, a gene, which is known to be transcriptionally activated by GLI2 (Regl *et al*, 2004). As treatment of *GLI1*-amplified RMS-13 cells with the hedgehog inhibitor cyclopamine was unable to induce changes in reporter activity in RMS-13 cells, these results suggest that BA inhibits hedgehog signalling directly on the level of GLI family transcription factors. This might be of enormous relevance, as a recent study reports on high *GLI1* expression levels in the majority of human RMS (Tostar *et al*, 2006). However, the underlying molecular mechanism for this activation is not entirely clear, as DNA amplifications at the *GLI1* locus has only been found in single RMS cases (Khatib *et al*, 1993; Goldstein *et al*, 2006) and *PTCH1* mutations are relatively rare in RMS (Calzada-Wack *et al*, 2002). Collectively, these data suggest that BA-induced apoptosis could be at least in part associated with the inhibitory effect of BA on hedgehog signalling and its target genes.

Altogether, our study represents the first proof-of-principle study of the effectiveness of BA on RMS-13 growth and hedgehog signalling *in vivo*. However, it seems that accelerated cell survival and activated hedgehog signalling are only part of the molecular composition of RMS-13 cells, as no regression was observed in BA-treated xenografts. Thus, other genetic lesions must be responsible for driving the exacerbated proliferation, which stays merely unchanged on BA treatment. It is highly probable that this characteristic feature of RMS cells could be effectively tackled by cytostatic drugs, thereby making combination therapies an attractive option to treat this deadly cancer. Our finding on the effective treatment of RMS-13 xenografts along with earlier *in vivo* data on BA treatment of melanoma, ovarian carcinoma, and prostate cancer (Pisha *et al*, 1995; Zuco *et al*, 2002; Chintharlapalli *et al*, 2007) strongly encourages such efforts.

## References

[bib1] Alakurtti S, Makela T, Koskimies S, Yli-Kauhaluoma J (2006) Pharmacological properties of the ubiquitous natural product betulin. Eur J Pharm Sci 29: 1–13 16716572 10.1016/j.ejps.2006.04.006

[bib2] Armistead PM, Salganick J, Roh JS, Steinert DM, Patel S, Munsell M, El-Naggar AK, Benjamin RS, Zhang W, Trent JC (2007) Expression of receptor tyrosine kinases and apoptotic molecules in rhabdomyosarcoma: correlation with overall survival in 105 patients. Cancer 110: 2293–2303 17896786 10.1002/cncr.23038

[bib3] Barlow JW, Wiley JC, Mous M, Narendran A, Gee MF, Goldberg M, Sexsmith E, Malkin D (2005) Differentiation of rhabdomyosarcoma cell lines using retinoic acid. Paediatr Blood Cancer 47: 773–784 10.1002/pbc.20650 16283617

[bib4] Beer C, Buhr P, Hahn H, Laubner D, Wirth M (2003) Gene expression analysis of murine cells producing amphotropic mouse leukaemia virus at a cultivation temperature of 32 and 37 degrees C. J Gen Virol 84: 1677–1686 12810861 10.1099/vir.0.18871-0

[bib5] Borgstrom P, Bourdon MA, Hillan KJ, Sriramarao P, Ferrara N (1998) Neutralizing anti-vascular endothelial growth factor antibody completely inhibits angiogenesis and growth of human prostate carcinoma micro tumours *in vivo*. Prostate 35: 1–10 9537593 10.1002/(sici)1097-0045(19980401)35:1<1::aid-pros1>3.0.co;2-o

[bib6] Breneman JC, Lyden E, Pappo AS, Link MP, Anderson JR, Parham DM, Qualman SJ, Wharam MD, Donaldson SS, Maurer HM, Meyer WH, Baker KS, Paidas CN, Crist WM (2003) Prognostic factors and clinical outcomes in children and adolescents with metastatic rhabdomyosarcoma – a report from the Intergroup Rhabdomyosarcoma Study IV. J Clin Oncol 21: 78–84 12506174 10.1200/JCO.2003.06.129

[bib7] Calzada-Wack J, Schnitzbauer U, Walch A, Wurster KH, Kappler R, Nathrath M, Hahn H (2002) Analysis of the PTCH coding region in human rhabdomyosarcoma. Hum Mutat 20: 233–234 10.1002/humu.9056 12204003

[bib8] Chintharlapalli S, Papineni S, Ramaiah SK, Safe S (2007) Betulinic acid inhibits prostate cancer growth through inhibition of specificity protein transcription factors. Cancer Res 67: 2816–2823 17363604 10.1158/0008-5472.CAN-06-3735

[bib9] Crist WM, Anderson JR, Meza JL, Fryer C, Raney RB, Ruymann FB, Breneman J, Qualman SJ, Wiener E, Wharam M, Lobe T, Webber B, Maurer HM, Donaldson SS (2001) Intergroup rhabdomyosarcoma study-IV: results for patients with nonmetastatic disease. J Clin Oncol 19: 3091–3102 11408506 10.1200/JCO.2001.19.12.3091

[bib10] Crist WM, Kun LE (1991) Common solid tumours of childhood. N Engl J Med 324: 461–471 1988832 10.1056/NEJM199102143240706

[bib11] Davis RJ, D'Cruz CM, Lovell MA, Biegel JA, Barr FG (1994) Fusion of PAX7 to FKHR by the variant t(1;13)(p36;q14) translocation in alveolar rhabdomyosarcoma. Cancer Res 54: 2869–2872 8187070

[bib12] Ehrhardt H, Fulda S, Fuhrer M, Debatin KM, Jeremias I (2004) Betulinic acid-induced apoptosis in leukemia cells. Leukemia 18: 1406–1412 15201849 10.1038/sj.leu.2403406

[bib13] Eichenmuller M, Gruner I, Hagl B, Haberle B, Muller-Hocker J, von Schweinitz D, Kappler R (2009a) Blocking the hedgehog pathway inhibits hepatoblastoma growth. Hepatology 49: 482–490 19177589 10.1002/hep.22649

[bib14] Eichenmuller M, von Schweinitz D, Kappler R (2009b) Betulinic acid treatment promotes apoptosis in hepatoblastoma cells. Int J Oncol 35: 873–879 19724925 10.3892/ijo_00000402

[bib15] Evangelista M, Tian H, de Sauvage FJ (2006) The hedgehog signalling pathway in cancer. Clin Cancer Res 12: 5924–5928 17062662 10.1158/1078-0432.CCR-06-1736

[bib16] Folkman J (2002) Role of angiogenesis in tumour growth and metastasis. Semin Oncol 29: 15–18 10.1053/sonc.2002.37263 12516034

[bib17] Fulda S, Friesen C, Los M, Scaffidi C, Mier W, Benedict M, Nunez G, Krammer PH, Peter ME, Debatin KM (1997) Betulinic acid triggers CD95 (APO-1/Fas)- and p53-independent apoptosis via activation of caspases in neuroectodermal tumours. Cancer Res 57: 4956–4964 9354463

[bib18] Fulda S, Jeremias I, Steiner HH, Pietsch T, Debatin KM (1999) Betulinic acid: a new cytotoxic agent against malignant brain-tumour cells. Int J Cancer 82: 435–441 10399962 10.1002/(sici)1097-0215(19990730)82:3<435::aid-ijc18>3.0.co;2-1

[bib19] Fulda S, Scaffidi C, Susin SA, Krammer PH, Kroemer G, Peter ME, Debatin KM (1998) Activation of mitochondria and release of mitochondrial apoptogenic factors by betulinic acid. J Biol Chem 273: 33942–33948 9852046 10.1074/jbc.273.51.33942

[bib20] Goldstein M, Meller I, Issakov J, Orr-Urtreger A (2006) Novel genes implicated in embryonal, alveolar, and pleomorphic rhabdomyosarcoma: a cytogenetic and molecular analysis of primary tumours. Neoplasia 8: 332–343 16790082 10.1593/neo.05829 PMC1592451

[bib21] Kappler R, Calzada-Wack J, Schnitzbauer U, Koleva M, Herwig A, Piontek G, Graedler F, Adamski J, Heinzmann U, Schlegel J, Hemmerlein B, Quintanilla-Martinez L, Hahn H (2003) Molecular characterisation of patched-associated rhabdomyosarcoma. J Pathol 200: 348–356 12845631 10.1002/path.1361

[bib22] Khatib ZA, Matsushime H, Valentine M, Shapiro DN, Sherr CJ, Look AT (1993) Coamplification of the CDK4 gene with MDM2 and GLI in human sarcomas. Cancer Res 53: 5535–5541 8221695

[bib23] Kwon HJ, Shim JS, Kim JH, Cho HY, Yum YN, Kim SH, Yu J (2002) Betulinic acid inhibits growth factor-induced *in vitro* angiogenesis via the modulation of mitochondrial function in endothelial cells. Jpn J Cancer Res 93: 417–425 11985792 10.1111/j.1349-7006.2002.tb01273.x PMC5927016

[bib24] Merlino G, Helman LJ (1999) Rhabdomyosarcoma – working out the pathways. Oncogene 18: 5340–5348 10498887 10.1038/sj.onc.1203038

[bib25] Mukherjee R, Jaggi M, Rajendran P, Siddiqui MJ, Srivastava SK, Vardhan A, Burman AC (2004) Betulinic acid and its derivatives as anti-angiogenic agents. Bioorg Med Chem Lett 14: 2181–2184 15081004 10.1016/j.bmcl.2004.02.044

[bib26] Muller CR, Paulsen EB, Noordhuis P, Pedeutour F, Saeter G, Myklebost O (2007) Potential for treatment of liposarcomas with the MDM2 antagonist Nutlin-3A. Int J Cancer 121: 199–205 17354236 10.1002/ijc.22643

[bib27] Newton WA, Gehan EA, Webber BL, Marsden HB, van Unnik AJ, Hamoudi AB, Tsokos MG, Shimada H, Harms D, Schmidt D, Ninfo V, Cavazzama AO, Gonzalez–Crussi F, Parham DM, Reiman HM, Asmar L, Beltangady MS, Sachs NE, Triche TJ, Maurer HM (1995) Classification of rhabdomyosarcomas and related sarcomas. Pathologic aspects and proposal for a new classification – an Intergroup Rhabdomyosarcoma Study. Cancer 76: 1073–1085 8625211 10.1002/1097-0142(19950915)76:6<1073::aid-cncr2820760624>3.0.co;2-l

[bib28] Pavlakovic H, Havers W, Schweigerer L (2001) Multiple angiogenesis stimulators in a single malignancy: implications for anti-angiogenic tumour therapy. Angiogenesis 4: 259–262 12197470 10.1023/a:1016045012466

[bib29] Pisha E, Chai H, Lee IS, Chagwedera TE, Farnsworth NR, Cordell GA, Beecher CW, Fong HH, Kinghorn AD, Brown DM, Wani MC, Wall ME, Hieken TJ, Das Gupta TK, Pezzuto JM (1995) Discovery of betulinic acid as a selective inhibitor of human melanoma that functions by induction of apoptosis. Nat Med 1: 1046–1051 7489361 10.1038/nm1095-1046

[bib30] Regl G, Kasper M, Schnidar H, Eichberger T, Neill GW, Philpott MP, Esterbauer H, Hauser-Kronberger C, Frischauf AM, Aberger F (2004) Activation of the BCL2 promoter in response to Hedgehog/GLI signal transduction is predominantly mediated by GLI2. Cancer Res 64: 7724–7731 15520176 10.1158/0008-5472.CAN-04-1085

[bib31] Roberts WM, Douglass EC, Peiper SC, Houghton PJ, Look AT (1989) Amplification of the gli gene in childhood sarcomas. Cancer Res 49: 5407–5413 2766305

[bib32] Rzeski W, Stepulak A, Szymanski M, Sifringer M, Kaczor J, Wejksza K, Zdzisinska B, Kandefer-Szerszen M (2006) Betulinic acid decreases expression of bcl-2 and cyclin D1, inhibits proliferation, migration and induces apoptosis in cancer cells. Naunyn Schmiedebergs Arch Pharmacol 374: 11–20 16964520 10.1007/s00210-006-0090-1

[bib33] Sarek J, Klinot J, Dzubak P, Klinotova E, Noskova V, Krecek V, Korinkova G, Thomson JO, Janost'akova A, Wang S, Parsons S, Fischer PM, Zhelev NZ, Hajduch M (2003) New lupane derived compounds with pro-apoptotic activity in cancer cells: synthesis and structure-activity relationships. J Med Chem 46: 5402–5415 14640549 10.1021/jm020854p

[bib34] Schmidt ML, Kuzmanoff KL, Ling-Indeck L, Pezzuto JM (1997) Betulinic acid induces apoptosis in human neuroblastoma cell lines. Eur J Cancer 33: 2007–2010 9516843 10.1016/s0959-8049(97)00294-3

[bib35] Schweigerer L, Neufeld G, Mergia A, Abraham JA, Fiddes JC, Gospodarowicz D (1987) Basic fibroblast growth factor in human rhabdomyosarcoma cells: implications for the proliferation and neovascularization of myoblast-derived tumours. Proc Natl Acad Sci USA 84: 842–846 2433691 10.1073/pnas.84.3.842 PMC304312

[bib36] Shapiro DN, Sublett JE, Li B, Downing JR, Naeve CW (1993) Fusion of PAX3 to a member of the forkhead family of transcription factors in human alveolar rhabdomyosarcoma. Cancer Res 53: 5108–5112 8221646

[bib37] Taipale J, Chen JK, Cooper MK, Wang B, Mann RK, Milenkovic L, Scott MP, Beachy PA (2000) Effects of oncogenic mutations in smoothened and patched can be reversed by cyclopamine. Nature 406: 1005–1009 10984056 10.1038/35023008

[bib38] Tostar U, Malm CJ, Meis-Kindblom JM, Kindblom LG, Toftgard R, Unden AB (2006) Deregulation of the hedgehog signalling pathway: a possible role for the PTCH and SUFU genes in human rhabdomyoma and rhabdomyosarcoma development. J Pathol 208: 17–25 16294371 10.1002/path.1882

[bib39] Visser M, Sijmons C, Bras J, Arceci RJ, Godfried M, Valentijn LJ, Voute PA, Baas F (1997) Allelotype of paediatric rhabdomyosarcoma. Oncogene 15: 1309–1314 9315099 10.1038/sj.onc.1201302

[bib40] Vousden KH, Lu X (2002) Live or let die: the cell's response to p53. Nat Rev Cancer 2: 594–604 12154352 10.1038/nrc864

[bib41] Zhang L, Yu D, Hicklin DJ, Hannay JA, Ellis LM, Pollock RE (2002) Combined anti-fetal liver kinase 1 monoclonal antibody and continuous low-dose doxorubicin inhibits angiogenesis and growth of human soft tissue sarcoma xenografts by induction of endothelial cell apoptosis. Cancer Res 62: 2034–2042 11929822

[bib42] Zuco V, Supino R, Righetti SC, Cleris L, Marchesi E, Gambacorti-Passerini C, Formelli F (2002) Selective cytotoxicity of betulinic acid on tumour cell lines, but not on normal cells. Cancer Lett 175: 17–25 11734332 10.1016/s0304-3835(01)00718-2

